# Emergency management of patients with ST-segment elevation myocardial infarction in Eastern Austria: a descriptive quality control study

**DOI:** 10.1186/s13049-018-0504-3

**Published:** 2018-05-09

**Authors:** Helmut Trimmel, Thomas Bayer, Wolfgang Schreiber, Wolfgang G. Voelckel, Lukas Fiedler

**Affiliations:** 1 Department of Anesthesiology, Emergency and Critical Care Medicine, General Hospital Wiener Neustadt, Corvinusring 3-5, A 2700 Wiener Neustadt, Austria; 2 Karl Landsteiner Institute of Emergency Medicine, General Hospital Wiener Neustadt, Corvinusring 3-5, A 2700 Wiener Neustadt, Austria; 3 Department of Internal Medicine II, General Hospital Wiener Neustadt, Wiener Neustadt, Austria; 4 Medical University Vienna, Vienna, Austria; 5 Department of Anesthesiology and Critical Care Medicine, AUVA Trauma Center Salzburg, Salzburg, Austria; 6 University of Stavanger, Network for Medical Sciences, Stavanger, Norway; 7 Paracelsus Medical University Salzburg, Salzburg, Austria

**Keywords:** ST-segment elevation myocardial infarction, Network, Time intervals, Pre-hospital management, Quality control study

## Abstract

**Background:**

Myocardial infarction is a time-critical condition and its outcome is determined by appropriate emergency care. Thus we assessed the efficacy of a supra-regional ST-segment elevation myocardial infarction (STEMI) network in Easternern Austria.

**Methods:**

The Eastern Austrian STEMI network serves a population of approx. 766.000 inhabitants within a region of 4186 km^2^. Established in 2007, it now comprises 20 pre-hospital emergency medical service (EMS) units (10 of these physician-staffed), 4 hospitals and 3 cardiac intervention centres. Treatment guidelines were updated in 2012 and documentation within a web-based STEMI registry became mandatory. For this retrospective qualitative control study, data from February 2012–April 2015 was assessed.

**Results:**

A total of 416 STEMI cases were documented, and 99% were identified by EMS within 6 (4.0–8.0) minutes after arrival. Median time loss between onset of pain and EMS call was 54 (20–135) minutes; response, pre-hospital and door-to-balloon times were 14 (10–20), 46 (37–59) and 45 (32–66) minutes, respectively. When general practitioners were involved, time between onset of pain and balloon inflation significantly increased from 180 (135–254) to 218 (155–348) minutes (*p* < .001). A pre-hospital time < 30 min was achieved in 25.8% of all patients during the day vs. 11.6% during the night (*p* < .001). Three hundred forty-five patients (83%) were subjected to primary percutaneous coronary intervention (PPCI), and 6.5% were thrombolysed by EMS. Pre-hospital complication rate was 18% (witnessed cardiac arrest 7%, threatening arrhythmias 6%, cardiogenic shock 5%). Twenty-four hours and hospital mortality rate were 1.2 and 2.8%, respectively.

**Discussion:**

Optimal patient care and subsequently outcome of STEMI is strongly determined by a short patient-decision time to call EMS and by the first medical contact to balloon time (FMCBT). Supra-regional networks are key in order to increase the efficacy and efficiency of health care. The goal of 120 min FMCBT was achieved in 78% of our patients immediately managed by EMS, thus indicating room for improvement.

**Conclusion:**

In conclusion, results from the Eastern Austrian STEMI network shed light on the necessity of increasing patient awareness in order to minimize any time loss derived by delayed EMS calls. Involvement of family physicians resulted in prolonged FMCBT. A stronger utilization of rescue helicopters could further improve the efficacy of this supra-regional network. Nevertheless PPCI rates, time intervals and outcome rates compare well with international benchmarks.

## Background

Acute, non-traumatic chest pain is one of the leading causes for EMS activation [1] and 15% of all missions are triggered by cardiac events [2]. In Austria, 20.000 patients per year suffer from an acute coronary syndrome (ACS) [3] comprising unstable angina pectoris, Non-ST-segment elevation myocardial infarction (NSTEMI), and ST-segment elevation myocardial infarction (STEMI). In order to assess the quality of emergency medical service (EMS) care, ACS is one of four accepted tracer diagnoses where appropriate management will most likely affect outcome [4–6]. Since pre-hospital mortality of STEMI patients is still high, timely identification and intervention is crucial [7, 8]. The European Society of Cardiology recommends that STEMI patients should be identified within 10 min after first medical contact, and, whenever possible, primary percutaneous coronary intervention (PPCI) should be initiated in less than 120 min. Any delay caused by a deferred EMS dispatch, any involvement of non-PCI-capable hospitals and emergency departments should be avoided [9, 10]. In order to match these requirements and to optimize pre-hospital medication, local STEMI networks should be established.

The Eastern Austrian STEMI Network was founded in 2007. Strict adherence to the consensus-based STEMI network treatment guidelines is obligatory for all EMS involved. Guidelines were revised in 2012, and Clopidogrel was replaced by Ticagrelor as first-line oral anticoagulant to be administered in combination with intravenous unfractionated heparin and salicylic acid.

In order to evaluate the efficiency and efficacy of our STEMI Network, we sought to assess all time intervals indicative for outcome, survival rates and possible complications.

## Methods

**Fig. 1:**
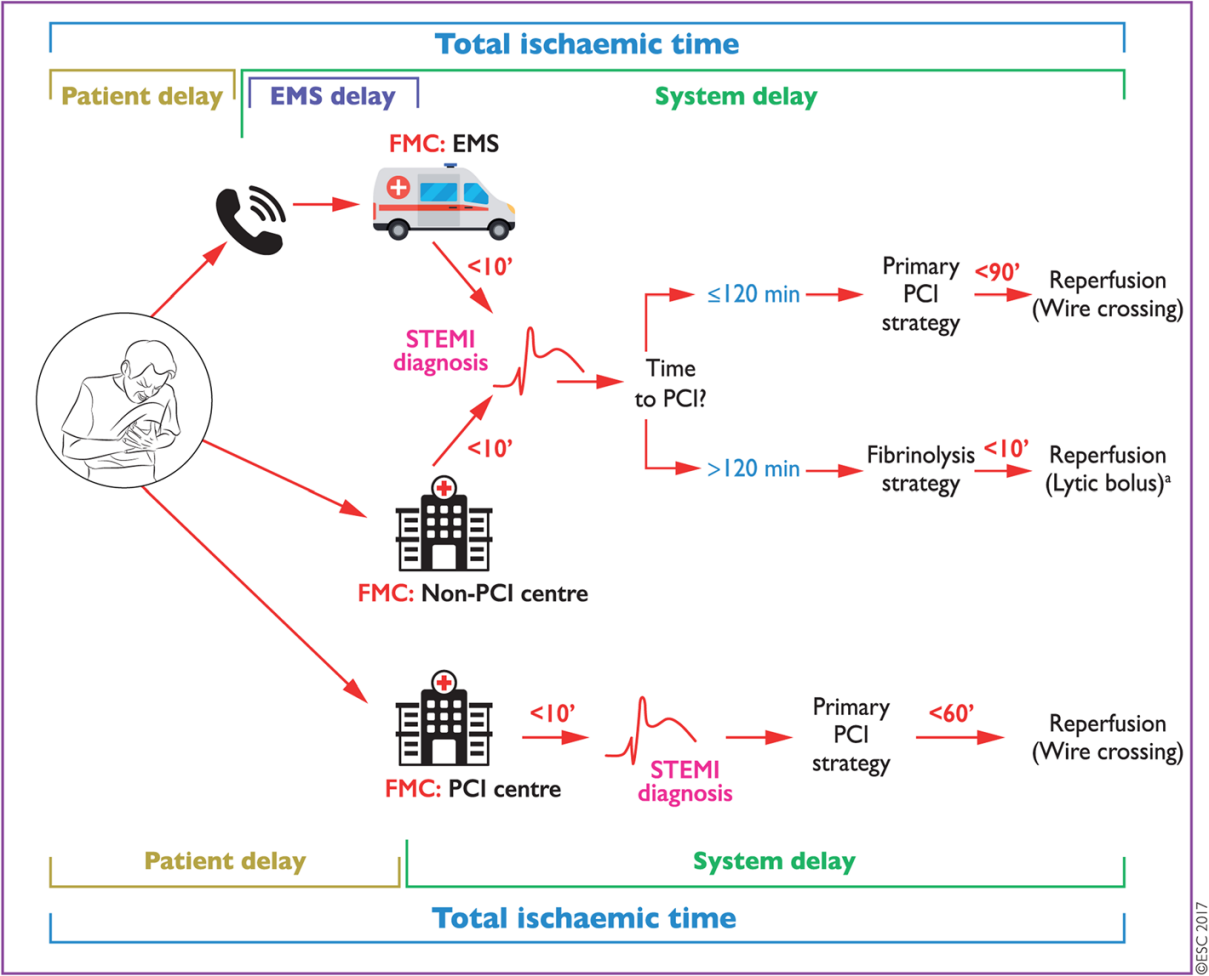
Prehospital reperfusion strategies (ESC Guidelines 2017). *Legend*: Modes of patient presentation, components of ischaemia time and flowchart for reperfusion strategy selection. EMS = Emergency Medical System; FMC = First Medical Contact; PCI = Percutaneous Coronary Intervention; STEMI = ST-segment elevation myocardial infarction. The recommended mode of patient presentation is by alerting the EMS (call national emergency number: 112 or similar number according to region). When STEMI diagnosis is made in the out-of-hospital setting (via EMS) or in a non-PCI centre, the decision for choosing reperfusion strategy is based on the estimated time from STEMI diagnosis to PCI-mediated reperfusion (wire crossing). System delay for patients alerting the EMS starts at the time of phone alert, although FMC occurs when EMS arrives to the scene. ´denotes minutes. aPatients with fibrinolysis should be transferred to a PCI centre immediately after administration of the lytic bolus. From: Eur Heart J. Published online August 26, 2017. doi:10.1093/eurheartj/ehx393. With permission of Oxford Academic Journals, obtained Jan 30, 2018 (License number 4278940402904)

**Fig. 2:**
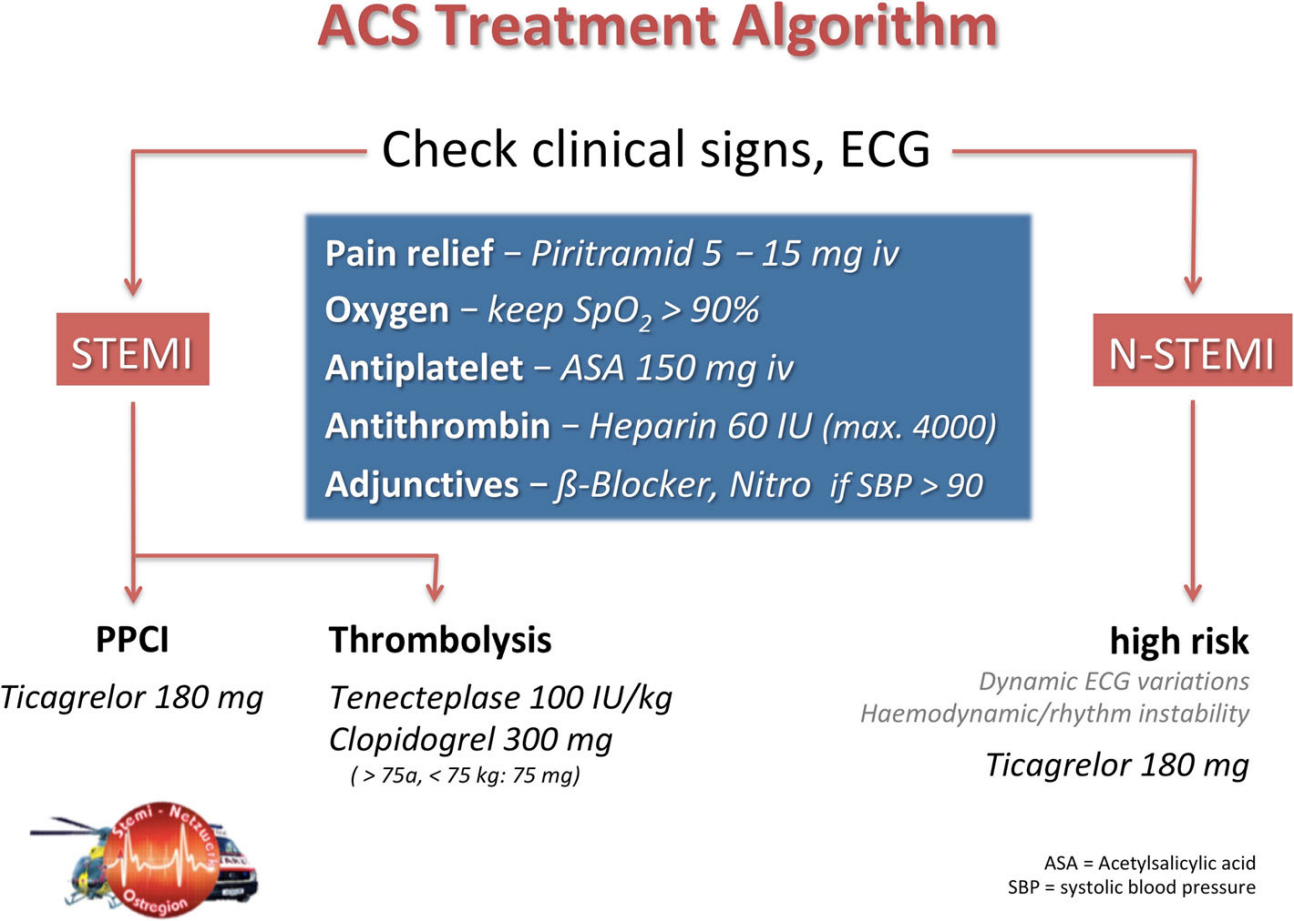
Prehospital algorithm for the treatment of ACS patients as defined by the Eastern Austria STEMI Network

The Eastern Austrian STEMI Network works to optimize emergency medical care of patients suffering from ACS across state boarders and to ensure the same level of therapy for all patients. The network serves a population of approx. 766,000 inhabitants within a region of 4186 km^2^. It now comprises 20 ground EMS units (8 of them physician-staffed), 2 helicopter emergency medical services (HEMS), 4 hospitals and 3 cardiac intervention centres with rotating 24/7 service. Transport distances to the PCI centre on duty may be up to 90 km. Whenever a STEMI is diagnosed, high priority is given to the time intervals and pre-hospital decision-making according to ESC guidelines [9] (Fig. 1). All guidelines and recommendations are re-assessed annually. Treatment and transport guidelines were updated in 2012. Based on current literature [11], the new platelet inhibitor Ticagrelor was added to the standard antithrombotic medication comprising unfractionated heparin and salicylic acid (Fig. 2). Documentation in a web-based STEMI registry (Survey Monkey®, Palo Alto, CA, USA) has been mandatory since 2012. Each data set must be completed when the patient is discharged from hospital, in order to document serious adverse events and outcome.

For this retrospective qualitative control study, data from February 2012–April 2015 was assessed. The study was approved by the ethical committee of the Medical University of Vienna (EK-ID number 1116/2016). Data obtained was handled according to current data protection guidelines.

Data was analysed employing MS Excel (Microsoft, Redmond, WA, USA) and SPSS 23 (IBM, NY, USA) and is presented as median and interquartile range or mean ± standard deviation whenever appropriate. For statistical tests ANOVA (analysis of variance), Kruskal-Wallis-Test or Chi^2^-Test, and Spearman correlation were used. A *p* value < 0.05 was considered indicative for significance.

## Results

During the study period, 416 cases were classified as STEMI in the pre-hospital setting, and subsequently transported to one of the 3 PCI centres. In 4 cases the prehospital assumed STEMI was not confirmed in hospital, and patients were diagnosed to suffer from Tako-Tsubo cardiomyopathy, pulmonary embolism or anaemia-driven cardiac ischemia, respectively.

### Primary findings

Patients were predominately male (73.8%) and younger (mean male vs. female age 61.7 ± 12.3 vs. 69.6 ± 13.9 years). STEMI was localized as anterior in 43.5% of cases, inferior in 41.1%, and lateral in 3.8%. In 11.6% of all cases, ST-segment elevation was found in both anterior and inferior or even more segments. 81.2% of the patients were found stable and normotensive, 11.3% hypotensive, and 2.9% required advanced treatment due to cardiogenic shock; 4.6% suffered cardiac arrest.

### Pre-hospital time intervals

**Fig. 3:**
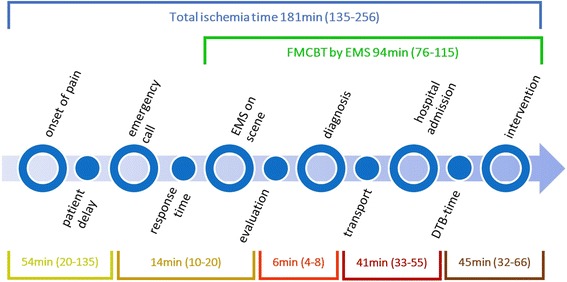
Timeline in STEMI patients. *Legend*: Critical points of time, related to the outcome of ACS patients. FMCBT = First medical contact to balloon time; EMS = emergency medical service; DBT = door to balloon time; ACS = acute coronary syndrome

**Fig. 4:**
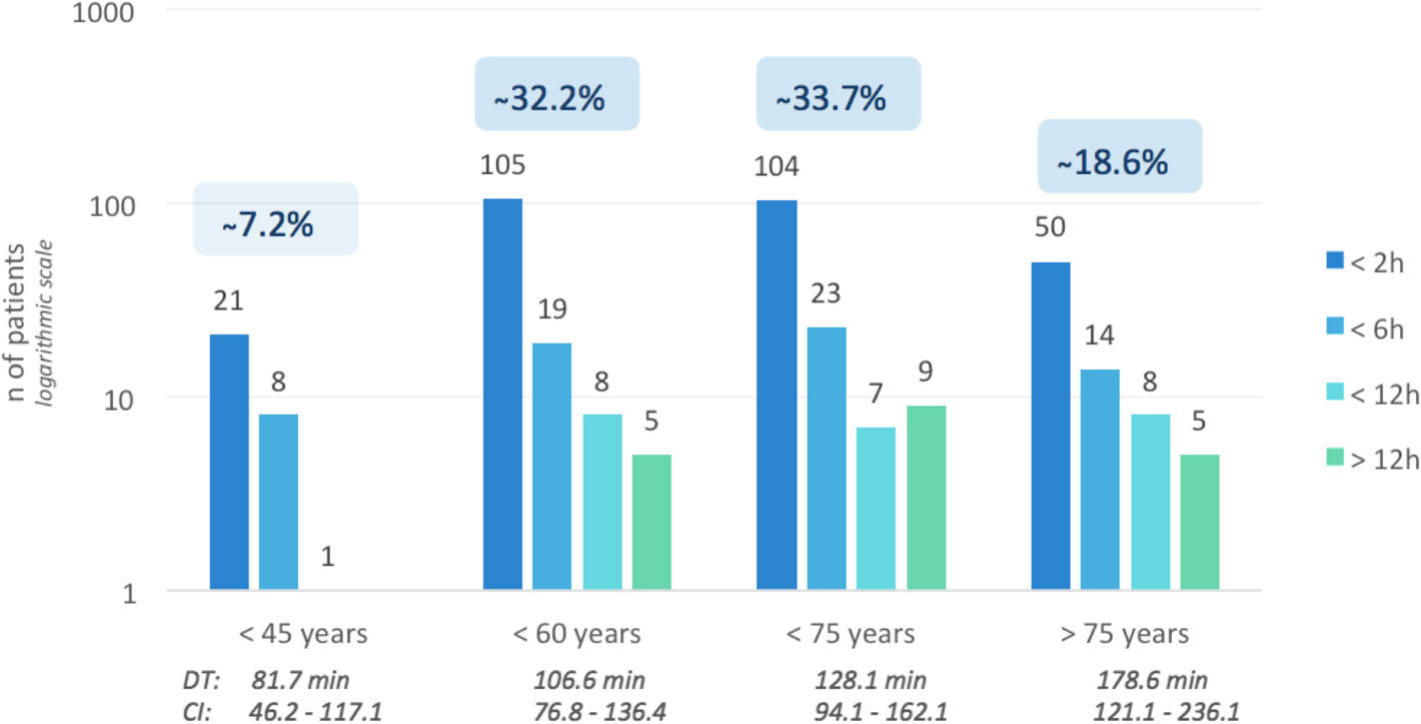
Patient delay, related to age. *Legend*: *n* = 387 (93% of 416 patients with STEMI; 29 (7%) were excluded due to lack of information on the onset of pain). Data are presented in groups reflecting the decision time: less than 2, 6, 12 or more than 12 h respectively (from onset of pain to emergency call, logarithmic plotting). Age groups: below 45, 60, 75 years or more than 75 years, respectively. DT = Mean value of delay, specified in minutes per age group; CI = Confidence Interval

The majority (72%) of all EMS missions for STEMI patients occurred between 06:00 and 20:00. Time intervals were further analysed in 387/416 patients (Fig. 3). Female patients hesitated longer than male patients to call EMS: 67 (IQR 28–171) compared with 45 (IQR 19–120) minutes, *p* < 0.003. Also, patients > 75 years waited longer than patients < 60 years and < 45 years: 75 (IQR 121–236) compared to 43.5 (IQR 77–136) and 35.5 (IQR 46–117) minutes (Fig. 4). In 28% of all cases, a general practitioner (GP) was involved, either at the patient’s home or in the doctor’s office. When patients consulted their family physician first and avoided direct EMS contact, a significant median time loss of 25.6 min until re-perfusion was noted compared with cases primarily managed by EMS. A pre-hospital time < 30 min was achieved in 25.8% of all patients during the day compared to 11.6% at night (*p* < 0.001).

### Pre-hospital therapy

STEMI network-specific management and pharmacotherapy algorithms are outlined in Figs. 1 and 2. In accordance with the ESC flowchart, pre-hospital thrombolysis was attempted in 27 cases (6.5%). Tenecteplase was administered within the first hour after onset of pain in 19 patients. Seven patients received Tenecteplase during on-going CPR, enabling return of spontaneous circulation on scene in 6 cases. Interestingly, EMS physicians considered pre-hospital thrombolysis indicated in 97 additional EMS missions, but noted contraindications in 35 patients. In 62 cases, pre-hospital thrombolysis was omitted based on the telephone advice of the PCI centre. All 62 patients were transported directly to the PCI centre, and a median FMCBT of 109 (IQR 83–134) minutes was achieved in this cohort.

### Primary percutaneous coronary interventions (PPCI)

381/416 patients (91.6%) underwent PCI. Direct patient handover in the catheter laboratory was performed in 13.7% of cases, but over 50% of all cases were admitted to either an emergency ward or an intensive care unit.

Median and interquartile door to balloon time was 45 (IQR 32–66) minutes. PPCI was successfully performed in 88.7%, while 4.6% underwent diagnostic angiography without further interventions. In 6.7% PCI was spared. In patients that had received pre-hospital thrombolysis, a rescue PCI was performed in 63% within 82 (IQR 62–144) minutes. The remaining thrombolysed patients were subjected to angiography within 10 days after admission.

### Ischemia time-intervals

Median FMCBT was 94.5 (IQR 76–115) minutes by EMS only. When a general practitioner was involved, FMCBT increased to 120 (IQR 110–151) minutes (*p <* 0.003).

In the patients handled primarily by EMS, the targets of FMCBT < 120 or even better < 90 min were reached in 78 and 46% of cases; in the GP first followed by EMS cohort, these targets were reached in 71 and 38% of cases (*p* < 0.05).

The median of total ischemia time (onset of pain until re-opening of the vessel) was 181.5 (IQR 135–256) minutes. As mentioned above, involvement of a GP resulted in an increased total ischemia time of 218 (IQR 155–347) minutes (*p <* 0.005).

### Complications

EMS physicians had to deal with complications in 23.8%, namely cardiac arrest (7%), arrhythmias (6%), cardiogenic shock (5.3%), as well as nausea, severe pain or respiratory problems (5.5%). In patients that had received pre-hospital thrombolysis (*N* = 27), re-perfusion arrhythmias were observed in 8 cases, and 5 patients developed cardiac arrest.

Ticagrelor was administered in 317/416 patients during the pre-hospital phase. Two of these patients (0.63%) had bleeding complications: one developed a minor hematoma at the radial puncture site; the other underwent angiography via femoral access and suffered from renal haematoma, presumably caused by the guide wire. The latter needed transfusion of two pRBCs.

### Outcome

At hospital admission 8.9% of all patients were classified as unstable, and 0.5% (N = 2) were transported during on-going CPR. Hospital outcome data was available for 394 patients. Five patients (1.2%) died within the first 24 h after admission, prior to or during PCI. Hospital mortality was 2.8% (*N* = 11). Seven of the deceased patients had to be resuscitated on scene or during transport, two were in cardiac shock or classified as unstable by the EMS physician, respectively. Only one of the non-survivors was classified as stable during pre-hospital care.

## Discussion

The European Society of Cardiology (ESC) recommends that a regional reperfusion strategy should be established to maximize efficiency of STEMI patient care [9]. In this retrospective quality control study, performance of the Eastern Austrian STEMI network was assessed over a 2-year period. We identified two major factors prolonging total ischemia time, namely a significant delay between onset of pain and EMS activation, and involvement of general practitioners. When compared with data from international STEMI registries or study groups, Eastern Austrian time intervals as well as PCI rates are competitive. Nevertheless, there is room for improvement such as better involvement of rescue helicopters and initiation of night flight programs. The latter is of specific interest, since 28% of STEMI cases occurred during night hours defined as the time between 20:00 and 06:00 and distances within the network might be as far as 90 km.

STEMI is a predominantly male disease with a male:female ratio of 2:1 to 3:1 [12]. Two other gender specific differences must be noted. First, male STEMI patients are younger when compared with female cardiac patients (62 compared to 70 years); and second, male patients activate EMS significantly earlier (45 compared to 67 min). The delay before calling EMS is also important in elderly patients, who might not experience the same pain level or interpret chest pain inappropriately: time from onset of pain to EMS activation was significantly longer in patients > 74 years than patients < 60 or < 45 years. Thus, creating better awareness for specific symptoms is of particular importance as required by the 2012 ESC guidelines: “*Patients with chest pain suggestive of MI should be directed through public awareness programs ...”* [13]*.* Once EMS is dispatched, the observed median response time is 14 min in our network and might be judged appropriate in a predominantly rural environment.

General practitioners play an important role in primary health care as gatekeepers and as first responders in rural areas. However, our data show that the 28% of the STEMI patients who were first seen by their family physician, either at home or in the doctor’s office, experienced a delay in activation of blue light and siren EMS; the two important time intervals, namely first medical contact to balloon (FMCBT) and total ischemia time were significantly prolonged (25.6 and 38 min, respectively). Thus, a median FMCBT < 120 min was achieved in 78% of immediate EMS vs. 71% practitioner first patients. When this ratio is compared with international FMCBT data there is room for improvement in our network [12, 14–18] because mortality rates will increase when delays cause an FMCBT > 1 h [14].

Another option to reduce FMCBT might be a better use of rescue helicopters for patient transport. Although the observed median transport times of 41 (33–55) minutes in our network are within international ranges, only 26% of all transports were shorter than 30 min during the daytime. This ratio further decreased to 12% at night. Helicopters were dispatched in only 17% of all cases during day, but are currently not available for night flying. Given the fact that the 3 PCI centres share the on duty rotation during the night, transport distances will increase and may reach up to 90 km. Thus employment of helicopters during night hours could contribute to reduced ischemia times [19, 20].

The door to balloon time (DBT) has been identified to have an impact on outcome [21, 22]. The observed median DBT of 45 min matches European standards [23], but half of all patients were still admitted at the emergency ward or intensive care unit first. Accordingly, better coordination and direct patient transfer to the catheter laboratory could lead to a reduction in DTB times in the PCI centres. Finally, the documented median total ischemia time of 181 min achieved in our network is comparable to European data derived from Germany (239 min) [15], Hungary (223 min) [16] and Sweden (175 min) [14]. In the APPOSITION-III trial, total ischemia times of 165, 270 and 360 min were reported for the Netherlands, Germany and France, respectively [18].

**Fig. 5:**
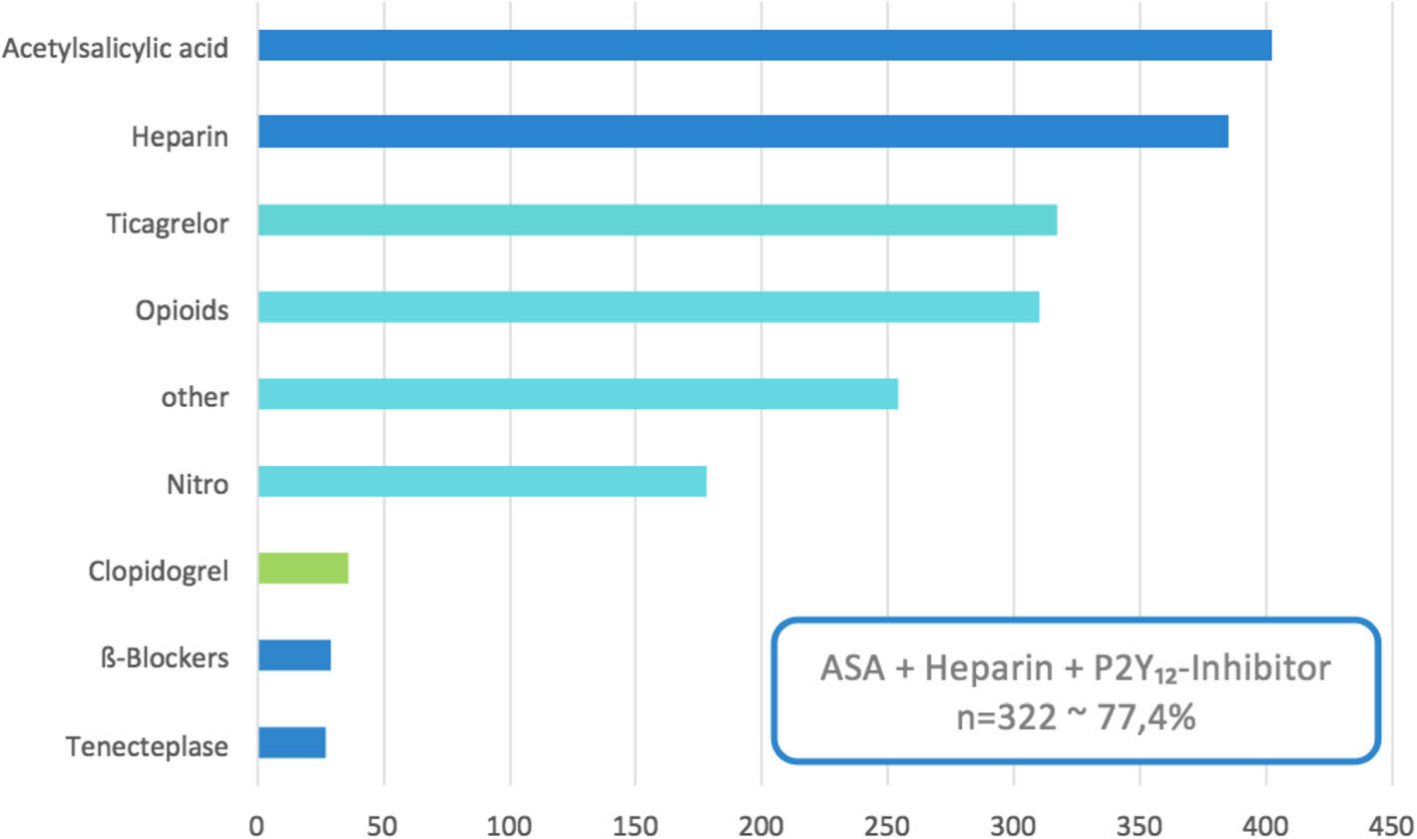
Prehospital medication, delivered by EMS. *Legend*: Medication delivered by EMS physicians (absolute numbers). npat = 416. ASA = Acetylsalicylic acid, P2Y12 Inhibitors: Ticagrelor, Clopidogrel

Besides the time-critical patient management comprising dispatch, response time, time to diagnosis, transport and door to balloon time (Fig. 3), early initiation and quality of medical care is required. On-site coagulation management might have an impact on outcome [11, 18]. In this regard, we consider two aspects of major importance. First, adherence to an accepted treatment algorithm, and second, communication loops with PCI centre cardiologists. We found both quality parameters adequately fulfilled in our network. The network-specific medication guideline, comprising unfractionated heparin, salicylic acid and Ticagrelor was successfully administered in 76% of all patients seen by EMS (see Fig. 5). Deviations from the guideline were justified by pre-existing patient medication such as warfarin. In particular, administration of 180 mg Ticagrelor orally was feasible and safe. During the entire study period, no life-threatening bleeding complication such as intra-cerebral haemorrhage was noted. Another possible life-saving intervention is the deliberate administration of thrombolytic drugs such as Tenecteplase. The importance of this therapeutic option is further supported by our findings. EMS physicians successfully performed an intravenous thrombolysis within 19 (IQR 11–27) minutes in 27 patients (6.5%). In an additional 97 missions, thrombolysis was considered but omitted. This was due to contraindications in 35 patients. In the remaining 62 cases, pre-hospital thrombolysis was withheld based on the telephone advice of the PCI centre. Interestingly, only 33 of the latter 62 patients underwent PPCI within the 120 min time span, so that on-scene thrombolysis would have been justified in 29 additional patients. Nevertheless, 95% of all patients diagnosed with STEMI were admitted at one of the three PCI centres in our network, and revascularization was attempted in 88.7%. Thus, our findings are similar to international data from France (87.6% PPCI, 12.4% thrombolysis) [24] and Germany (85.6% PPCI, 4.6% thrombolysis) [24].

In critical patients with life-threating arrhythmias, shock or cardiac arrest, immediate and qualified medical help is critical. Our EMS personnel were able to stabilize 7/12 patients with cardiogenic shock, and to achieve return of spontaneous circulation in 17/19 cardiac arrest patients. At hospital admission, 90% of all patients were considered haemodynamically stable. The first 24 h mortality was 1.2%, and hospital mortality was 2.8%. The observed mortality rate might be considered as a key indicator for the efficacy of our network. Heller [25] and Schmidt [26] reported hospital mortalities of 13.9 and 14.8% respectively in patients with acute myocardial infarction (AMI) in 2004–2008; Radovanovic reported hospital mortality of 5.5% for men and 6.9% for women for STEMI patients in 2017, enrolled in the Swiss nationwide registry (AMIS Plus) [27]. Thus, there has been a notable general reduction in hospital mortality in Europe during the last decades.

However, some limitations of our study must be noted. First, this is a purely retrospective study with data derived from an EMS-driven registry. Accordingly, data quality is strongly influenced by the documentation quality. Second, primary cardiac arrest patients were not included in this study, although STEMI or ACS might have been the reason for cardiac arrest. To our knowledge, cardiac arrest patients are typically not included in STEMI or ACS registries [28]. Finally, we did not have exact data on the time span between onset of pain and first medical contact for some of our patients who consulted general practitioners or family doctors; thus, we excluded those patients from further time analysis.

## Conclusion

In conclusion, results from the Eastern Austrian STEMI network shed light on the necessity of creating patient awareness in order to minimize any time loss derived by delayed EMS calls. Involvement of family physicians resulted in prolonged FMCBT. Although PPCI rates, time intervals and outcome rates match with international benchmarks, the efficacy of a supra-regional STEMI network with long transport distances could be improved when the best transport mean is employed deliberately for every patient. Thus further evaluation of the potential role of HEMS in STEMI networks is warranted.
